# Cold-Plasma-Driven Ammonia Synthesis over Porous Silica: The Role of the Morphology

**DOI:** 10.1021/acsmaterialsau.4c00159

**Published:** 2025-01-14

**Authors:** Fnu Gorky, Vashanti Storr, Jacek B. Jasinski, Maria L. Carreon

**Affiliations:** † Ralph E. Martin Department of Chemical Engineering, University of Arkansas, 3202 Bell Engineering Center, Fayetteville, Arkansas 72701-1201, United States; ‡ Conn Center for Renewable Energy Research, JB Speed School of Engineering, University of Louisville, Louisville, Kentucky 40292, United States

**Keywords:** nonthermal plasma, ammonia synthesis, mesoporous silica, macroporous silica, plasma catalysis

## Abstract

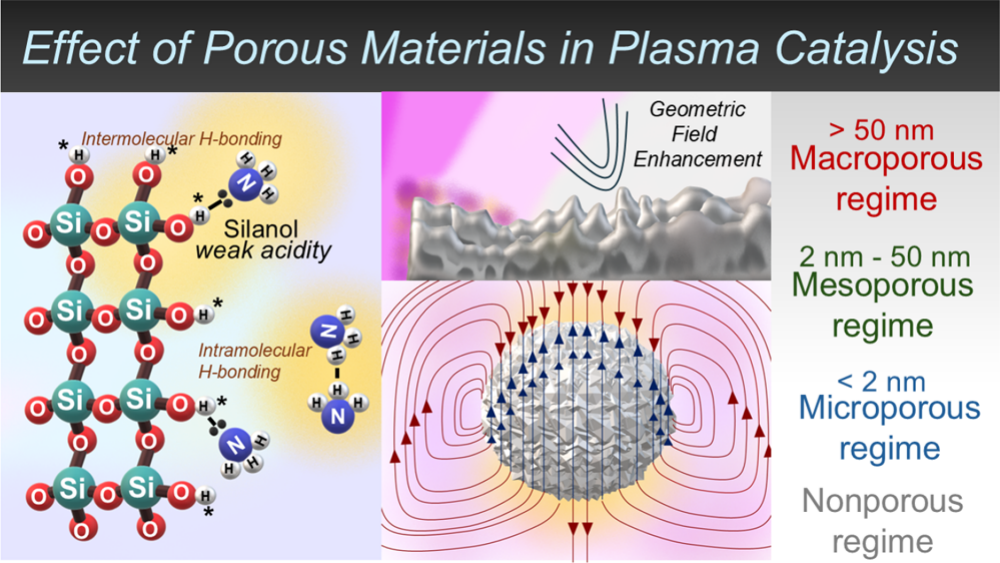

Nonthermal plasma (NTP) has opened unexplored routes for small-scale, decentralized ammonia production. However, understanding the ammonia formation pathways when employing NTP-driven processes is challenging due to the complex nature of the process. In this work, we report the effects of the morphology and textural properties on ammonia production. Herein, we explore mesoporous and macroporous regimes in silica. Performance of mesoporous silica with a gyroid morphology displays the highest ammonia production rate of 160.7 μmol/min·g-cat at a plasma power of 15 W. The findings from this work provide insights into tailoring porous structures and morphology for ammonia production powered by NTP. This work presents significant progress in the development of earth-abundant materials, achieved by tailoring their morphology and porous structure to improve their efficiency in plasma ammonia production.

## Introduction

Ammonia is an important building block for fertilizers and for the production of many other chemical processes.^1,2^ Recently, it has also been proposed as a sustainable fuel for both mobile and remote applications.^3^ Additionally, ammonia shows promise as a hydrogen carrier, offering a lower cost per unit of stored energy. For instance, 182 days of ammonia storage can cost only $0.54 per kg-H_2_, compared to almost $15 per kg-H_2_ for pure hydrogen storage.^4^ Ammonia also has a high volumetric energy density (7.1–2.9 MJ/L), added to widespread production, handling, and distribution capacity, which could further enhance its commercial viability.^5^ Therefore, long-sustained ammonia production can be foreseen.^6^ Currently, ammonia production predominantly occurs through the Haber–Bosch (H–B) process, which is often operated under harsh conditions, such as high temperature (650–750 K) and pressure (50–200 bar).^7^ Given the energy and reaction condition requirements, the H–B process is economically feasible only in large-scale facilities that require significant capital investments and a reliable supply of continuous electricity to maintain ongoing operations. Therefore, developing simplified alternatives to the H–B process that can operate under milder conditions and make use of intermittent electricity—renewable sources—represents a crucial step toward enabling small-scale, decentralized ammonia production. The application of plasma technology for ammonia production has emerged as a promising prospect.^8,9^ Nonthermal plasma (NTP)-driven ammonia synthesis can be operated at atmospheric pressure and low temperatures (<300 °C).^10,11^ In NTP, electrons are highly energetic with a temperature in the range of 10^4^–10^5^ K (1–10 eV), while the bulk gas temperature remains as low as room temperature.^12−14^ Instead of increasing the bulk temperature as in thermal processes, free electrons in NTP can initiate reactions. This approach allows for the conversion of nitrogen and hydrogen into ammonia under milder bulk conditions.^1,15^ Previous studies from our group have also indicated that the combination of plasma and selected materials can result in the efficient synthesis of ammonia.^16^ More importantly, plasma-based reaction systems offer great flexibility, safety, and fast on/off operations. Such advantages place NTP as a high-potential technology for decentralized small-scale ammonia synthesis powered by intermittent renewables, which increases the overall economic capability of the plasma process.

Nonetheless, NTP-mediated ammonia production is currently dealing with two major challenges: (i) the complexity of plasma–catalyst interactions and (ii) the plasma-induced reverse reactions (in situ ammonia decomposition) that take place during ammonia formation reactions concomitantly.^17−19^ The first obstacle leads to the lack of fundamental understanding of the plasma–catalyst synergism, which is crucial for optimizing and deploying the ammonia production process driven by NTP. To address this gap, it is essential to establish solid foundational knowledge that allows for the rational selection of suitable materials. The second challenge, on the other hand, reduces the ammonia product yield significantly. Researchers have indicated that the decomposition of freshly generated ammonia takes place by electron impact during microdischarges.^20−23^ Moreover, studies also reveal that the presence of porous materials with a certain morphology can limit the in situ decomposition of ammonia. For instance, our recent work confirmed that tailored geometry, i.e., the octahedral shape of the CC3 crystal^24^ or the pore size of mesoporous SBA-15,^25^ can offer protection from in situ ammonia decomposition, improving ammonia production rates. In terms of materials selection, solid oxides are considered more robust and affordable relative to their metal-based counterparts.^26^ Furthermore, solid oxides possess flexible textural and morphological properties that can be tailored for various reactions. Silica is among such solid oxides and has recently emerged as a potential outstanding candidate for plasma-assisted ammonia synthesis. Silica oxide offers a more abundant and economically viable alternative to the precious metals traditionally used in catalysis, such as platinum, palladium, and rhodium, which are commonly employed in thermal catalysis. However, recent work from our group observed that the performance and effects observed in thermal catalysis do not translate directly to plasma systems.^16^ This emphasizes the importance of identifying materials that are better suited to plasma environments. Furthermore, recent studies highlight a growing concern: precious metals (Au, Pt, Pd) face potential scarcity in the coming century due to limited reserves and rising demand across various industries, including energy storage.^27^ To avoid potential supply chain challenges, there is a need to look for sustainable alternative materials that can drive key processes without the exhaustion of critical resources. Silica oxide is a promising alternative that is not only earth-abundant but also exhibits properties that can enhance ammonia synthesis in plasma. Silica possesses low electronegativity (about 3.38) and low electrical resistivity that can lead to more stable and uniform plasma discharges.^25^ Our recent DFT calculations^16^ and experimental results^24^ have revealed that a desirable catalyst for ammonia synthesis is one that can delay the recombination of adsorbed H* into H_2_, enabling it to instead bind to adsorbed atomic nitrogen to form NH*. Also, it is necessary to prevent the in situ decomposition of ammonia due to plasma exposure. This can be achieved with the use of porous materials with high surface areas and porosity, which can promote the mass transfer of guest–product species.^28^

Numerous studies have demonstrated the superior performance of porous materials in various applications. For instance, in 2017, Peng’s work on Ru/MCM-41^29^ and our group work in 2018 on Ni-MOF-74^30^ experimentally revealed enhanced ammonia synthesis, providing critical motivation for further research. Building on this foundation, our group analyzed different porosity regimes from nonporous, microporous, and mesoporous in SBA-15^31^ in 2021. Recent studies reveal the significance of porosity^18,32,33^ and morphology^34−36^ in plasma-driven reactions. However, the intricate mechanisms by which the effect of morphology and porosity exist remain largely unexplained.

Herein, this work reports NTP-driven ammonia synthesis over silicas with different morphologies, including spheres and gyroids with various pore regimes, including mesopores and macropores. Among all morphologies, the spherical structure is regarded as the most stable in the literature.^37^ Spherical structures offer high mechanical strength, create shorter pathways for the diffusion of species, enhance dispersion through the stabilization of electrostatic charges, and feature a high surface-area-to-volume ratio.^37^ On the other hand, gyroid structures, with their triply periodic, three-dimensional, and interconnected pore networks, provide unique advantages for ammonia synthesis. Their large surface area and uniform pore size distribution enable efficient mass transfer and diffusion of reactants and products, while their intricate architecture promotes a high degree of stability.^38−40^ These advantages make them well-suited as materials for chemical reactions and explain our motivation for exploring this morphology. Herein, the influence of the morphology and surface area of the silica on plasma-driven ammonia production is addressed thoughtfully. The findings from this work aim to provide significant insights into abundant earth-tailored materials for plasma ammonia synthesis.

## Experimental Section

### Material Preparation

#### Mesoporous Silica Sphere

To prepare spherical mesoporous silica, cetyltrimethylammonium bromide (CTAB) was added to a solution consisting of *N*,*N*-dimethylformamide (DMF), NaOH, and deionized water. The solution was stirred for 30 min before adding TEOS and kept stirring at 40 °C for 24 h. The precursor solution was aged in a Teflon autoclave at 80 °C for 12 h. The white solid obtained was calcined at 500 °C with a heating rate of 1 °C/min for 4 h in air.

#### Mesoporous Silica Gyroid

Mesoporous silica with a gyroid morphology was synthesized via the sol–gel method, modified from elsewhere.^41^ The process was initiated by prehydrolyzation of TEOS in an ethanol solution by acid catalysis to obtain an oligomeric silica solution, which was added dropwise to a solution of P123 triblock copolymer and electrolyte in water and ethanol magnetically stirred at room temperature for 4 h. The final molar composition of the mixture was 1.0 TEOS:0.03 P123:1.0 MgSO_4_: 65 H_2_O:0.005 HCl:40 ethanol. The mother gel was aged for 48 h at 30 °C to maintain the interconnection of the sol–gel network. The solid products obtained, after being dried at 50 °C overnight and centrifuged three times with deionized water at 5000 rpm for 10 min to remove electrolytes, were further dried at 80 °C overnight. The solid products obtained were calcined to completely remove the block copolymer species in an oven at 500 °C at a 1 °C/min ramp for 12 h in air.

#### Macroporous Silica Sphere

Monodispersed macroporous silica was purchased from CD Bioparticles, Shirley, New York (Catalogue number: DNG-GS053).

### Material Characterization

The morphology of the silicas was inspected by the FEI Nova Nanolab 200 Dual-Beam system, equipped with high-resolution field-emission gun analytical scanning electron microscopy (SEM) at an acceleration voltage of 15 kV. Transmission electron microscopy (TEM) analysis was also performed using an FEI Co. Tecnai G2 F20 S-Twin 100 kV field-emission scanning transmission electron microscope. To prepare the TEM specimens, the samples were first diluted in methanol, sonicated, and then dispersed on standard Formvar carbon-on-copper 300 mesh TEM grids via a micropipette.

The prepared samples were degassed at 120 °C under vacuum for 12 h before nitrogen physisorption isotherms were conducted at 77 K (ASAP 2020 Plus +3 Flex, Micromeritics) to determine Brunauer–Emmett–Teller (BET) surface area, pore volume, and pore size of the fresh materials.

### Reactor Setup

Silicas with different porosities were tested in an in-house-built atmospheric dielectric barrier discharge (DBD) reactor.^15,31,42^ For the catalytic tests in this reactor, N_2_ and H_2_ cylinders were connected to the reactor using mass flow controllers. The reactions were carried out at a total flow rate of 25 sccm with various nitrogen and hydrogen feed ratios (1:1, 1:2, and 1:3) (N_2_/H_2_). The plasma power varied from 2.5 to 15 W with a 25 ± 0.5 kHz frequency, and separate experiments with frequency were performed ranging from 20 to 27 kHz. A wire from Midwest Tungsten Service was employed as an inner electrode. The outer electrode was made of tinned copper mesh acting as the ground electrode. The electrical characterization was carried out by measuring the applied voltage to the reactor by employing a high-voltage probe (Tektronix P6015A). The grounded electrode was connected via a 9.83 nF capacitor, which was in series with a 50 Ω resistor. The other terminal of the resistor, along with the ground of the high-voltage probe, was connected to the plasma power supply ground. The charge (*Q*) was calculated using the equation *Q* = *C* × *V*, where *C* = 9.83 nF is the capacitance and *V* is the voltage across the capacitor. The two probes were connected to an oscilloscope (Tektronix TDS2014C). The capacitor was connected to the reactor in series with the ground electrode. The high-voltage power supply was connected to the reactor using a litz wire and alligator clips. The inner electrodes were placed at the center of the quartz tube with an ID of 4 mm and an OD of 6.35 mm. The discharge gap for the in-house DBD was 0.2 mm. The gases collected from the reactor exit were sent to an online gas chromatograph to determine the ammonia synthesis rate. The quantification was performed using an Agilent 8860A + SRI 8610C gas chromatography system with an Agilent HP-PLOT U column (30 m × 0.320 mm × 10 μm) and an SRI MXT-U Bond capillary column (30 m × 0.53 mm × 20 μm), with hydrogen as the carrier gas. The distance between the reactor exit and the gas chromatography (GC) inlet was 37 cm (14 in.). Ammonia production experiments were conducted using gas chromatography, with a gas sampling time of 3 min. To address the limitation of this sampling time for desorption studies, a hand-held ammonia (NH_3_) gas detector (model: 0–5000 ppm, ATO) with a rapid monitoring time of 5 s was employed. The DBD reactor had a total volume of 1.29 mL, and the residence time with packed silica was calculated to be 2.78 s.

### Emission Spectroscopy

The light emitted from the discharge was led through an optical system. The measurements were recorded by using a dual-channel UV–vis–NIR spectrophotometer in scope mode (Avantes Inc., USB2000 Series). The spectral range was from 200 to 1100 nm, using a line grating of 600 lines/mm and a resolution of 0.4 nm. A bifurcated fiber optic cable with 400 μm was employed. For accuracy, the integration time was set at 1 s, with 50 averaging times.

## Results and Discussion

### Material Characterization

**Figure 1:**
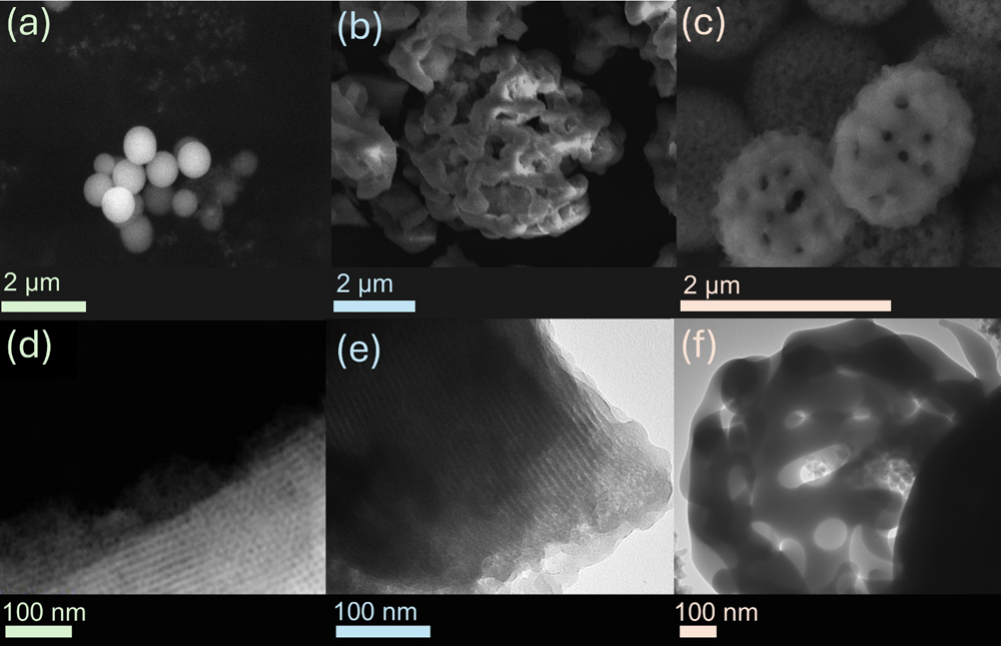
(a) SEM image of fresh mesoporous silica sphere, (b) SEM image of fresh mesoporous silica gyroid, (c) SEM image of fresh macroporous silica sphere, (d) TEM image of fresh mesoporous silica sphere, (e) TEM image of fresh mesoporous silica gyroid, and (f) TEM image of fresh macroporous silica sphere.

Figure 1 presents the morphology of all the prepared silica materials, including a mesoporous silica sphere and gyroid and a macroporous silica sphere. SEM images of all materials are presented in Figure 1. Figure 1a displays the presence of a well-defined mesoporous silica sphere. Figure 1b exhibits a three-dimensional mesoporous silica gyroid structure with numerous channels and cavities, while Figure 1c displays a macroporous silica sphere with noticeable pores. To gain a better understanding of the silica morphology, we also performed TEM. The TEM images (Figure 1d,e) of the mesoporous silica sphere and gyroid show a homogeneous particle size distribution. The macroporous silica sphere exhibits a homogeneous particle size distribution of approximately 100 nm (Figure 1f), confirming its identity as a macroporous material. Such a difference in the materials’ morphology is crucial for chemical reaction performance as it determines the electrical properties of the plasma and the associated molecular reaction rate, thereby altering the plasma catalytic material.^14^ The following sections will verify the effects of these morphological differences on catalytic performance.

### Textural Properties

**Figure 2:**
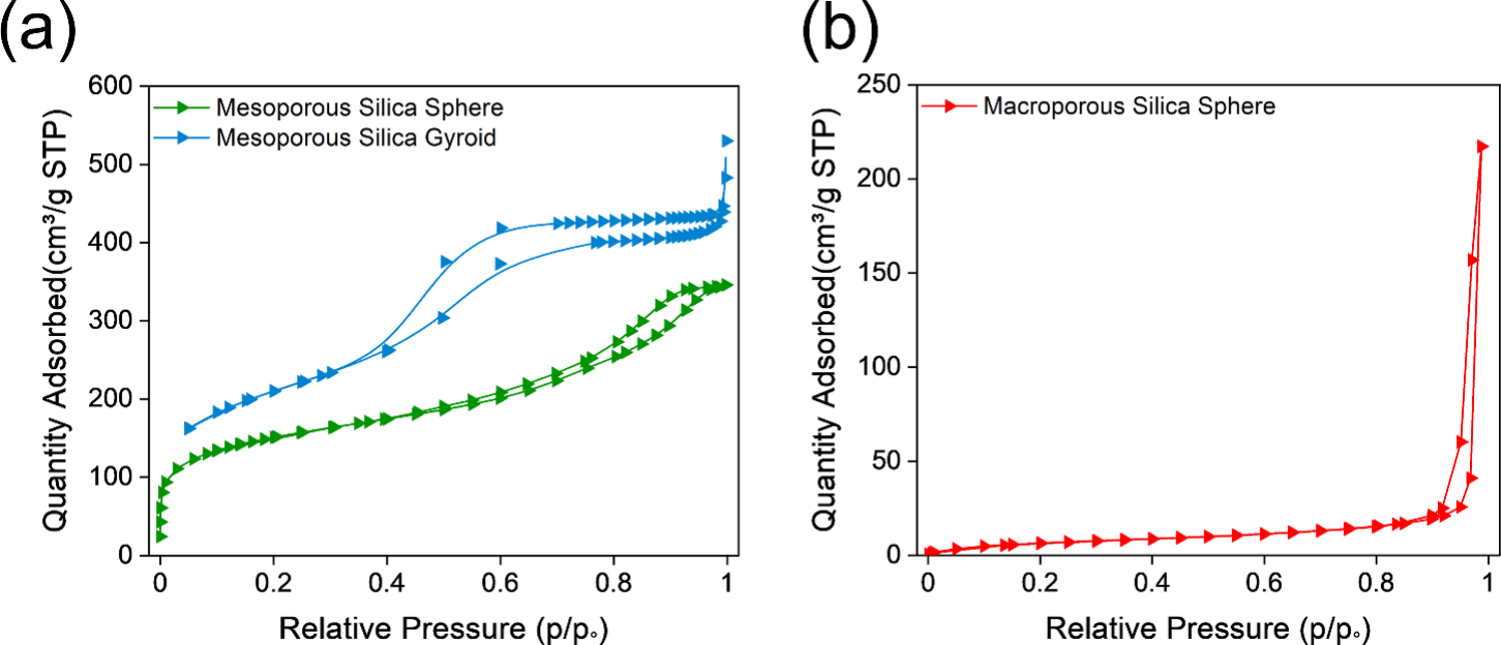
N_2_ adsorption–desorption isotherm curves of fresh materials: (a) Mesoporous silicas and (b) macroporous silica sphere.

N_2_ adsorption–desorption characterization was performed for all of the prepared silicas, and the results are shown in Figure 2. The N_2_ adsorption–desorption curve of the mesoporous silica sphere displays a type IV isotherm, affirming the presence of a mesoporous structure. It exhibits a hysteresis loop at higher pressures, indicating capillary condensation within the pores. This is classified as IUPAC Type H1, meaning the material has uniform, cylindrical pores.^43^ The mesoporous silica gyroid exhibits a type IV isotherm with an inclined mound-shaped hysteresis, classified as IUPAC H2. Furthermore, the N_2_ adsorption–desorption curve of the macroporous silica sphere shows a type III isotherm corresponding to macroporous materials.

The specific surface areas of all of the prepared materials are detailed in Table 1. Among the mesoporous silicas, the mesoporous silica gyroid (723 m^2^/g) shows a larger surface area compared to that of its spherical counterpart (521 m^2^/g). The macroporous silica sphere has a surface area of 19.8 m^2^/g, which is significantly lower than those of the other materials. According to IUPAC, materials are classified based on pore size: microporous (pore diameters <2 nm), mesoporous (2–50 nm), and macroporous (>50 nm).^43^ The macroporous silica sphere exhibits a pore size of 98 nm, which is typical for macroporous materials. The average pore size measured for the mesoporous silica sphere (3.92 nm) and mesoporous silica gyroid (3.26 nm) falls within the range expected for mesoporous materials.

**Table 1:** Textural Properties of All Prepared Materials

silica material	morphology	surface area	±	pore size	±
		m^2^/g		nm	
macroporous	sphere	19.80	4.58	98	
mesoporous	sphere	521		3.92	
mesoporous	gyroid	723.78	5.73	3.26	0.78

In this study, we aim to compare the ammonia synthesis rates of the mesoporous silica sphere, mesoporous silica gyroid, and macroporous silica sphere. A key consideration in our analysis is the significant difference in surface area among these structures, i.e., 521 m^2^/g for mesoporous silica sphere, 723 m^2^/g for mesoporous silica gyroid, and 19.8 m^2^/g for macroporous silica sphere. The variation in isotherm types reflects distinct adsorption and desorption profiles, as well as differing kinetics for each pore regime. Normalizing the data would not be objective, as the materials fall within distinct pore regimes, and such a comparison could lead to misleading interpretations of their performance. Therefore, we will present the absolute data for a clearer assessment of the differences in ammonia synthesis rates based on each material’s unique structural characteristics.

### Electrical Characterization

**Figure 3:**
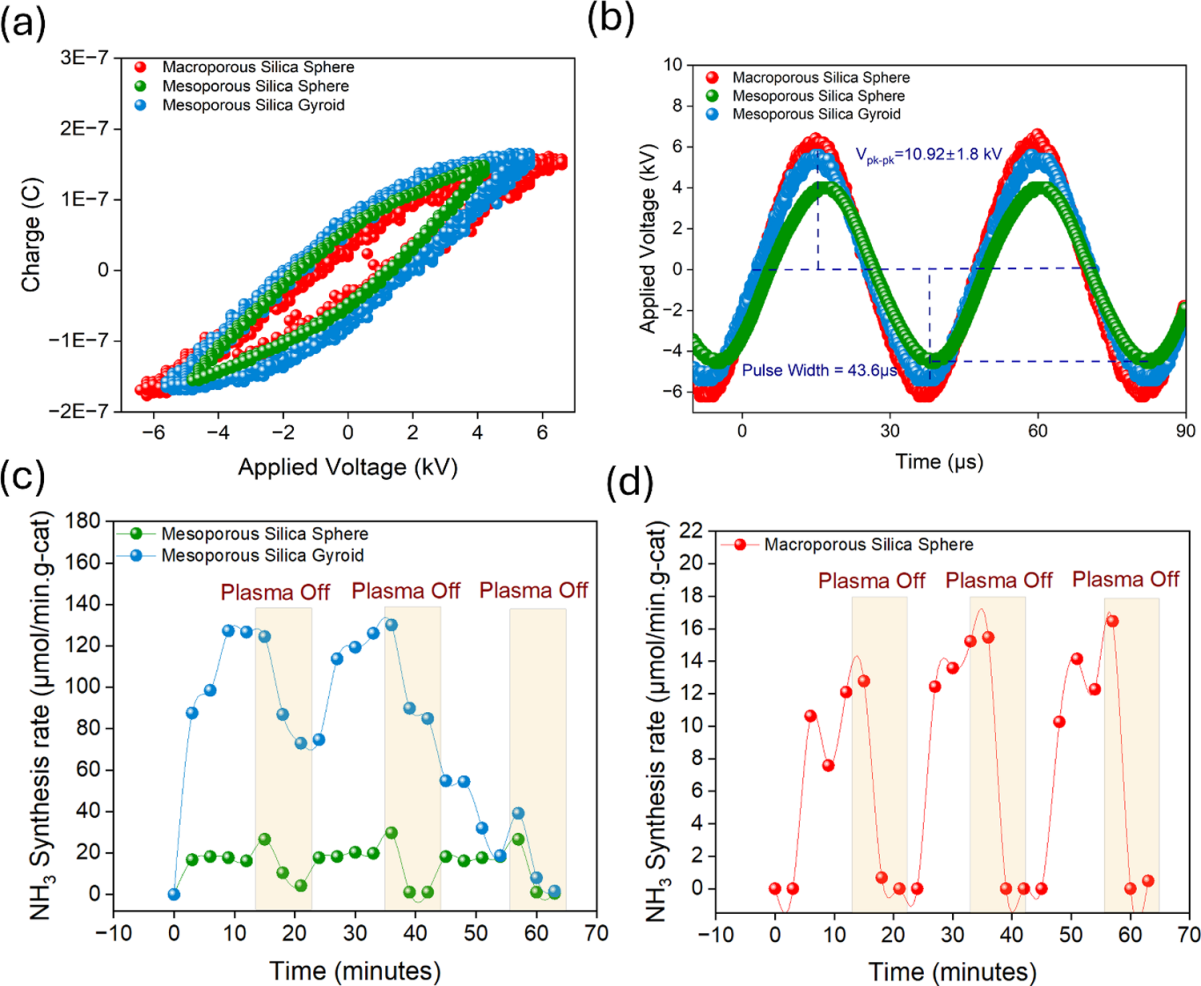
(a) Electrical characterization (applied voltage vs. charge); (b) discharge voltage; (c) plasma catalytic pulsing (plasma on/off) for mesoporous silica at a hydrogen rich feed (1:3) N_2_/H_2_ ratio, 25 sccm total flow rate, and 15 ± 0.2W plasma power; and (d) plasma catalytic pulsing (plasma on/off) for macroporous silica.

It is important to characterize the electrical properties of all prepared silica materials in the plasma environment to provide a better understanding of the influence of the morphology on the discharge and, hence, the impact on ammonia production rate. Figure 3a shows the voltage–charge curve with a well-defined Lissajous loop for all the prepared silica materials under plasma exposure. The electrical power was calculated by multiplying this value by the waveform frequency (AC coupling). The area of the Lissajous curve represents the energy dissipated by the discharge period. The largest dissipated energy was observed from the presence of the mesoporous silica gyroid, followed by the mesoporous silica sphere. As shown in Figure 3a, the shape of the Lissajous curve varies with each material packed. Although the applied power is consistently maintained at 15 W, the applied voltage differed for each material. A similar behavior was observed by Ndayirinde et al.,^44^ where differences in material composition affected the plasma characteristics. In our study, however, all of the employed materials are silicas, with variability arising from differences in morphology. This suggests that the observed changes in plasma behavior can be attributed to the morphological differences. The voltage–time graph (Figure 3b) exhibits an average discharged pk-to-pk voltage of 10.92 kV with a pulse width of 43.6 μs.

In this work, we also tested plasma pulsing (plasma on/off) to gain insights into the adsorption and desorption behavior of ammonia with various silica materials (Figure 3c,d). Pulsing-based experiments have been previously reported by our group^45−47^ and in the literature.^48−50^

This study is important for understanding how NTP reactors effectively pair with renewable energy sources for ammonia production in decentralized locations. Renewable energy sources, such as solar power, inherently experience variability due to factors such as time of day and weather conditions. By integrating pulsing NTP technology, we can optimize ammonia production to harness these intermittent energy supplies.

The results from the pulsed experiments show that the morphology and porous structure influence the desorption of ammonia. Specifically, the mesoporous silica gyroid exhibits the highest amount of desorbed ammonia after the plasma is switched off. This indicates that a significant portion of the produced ammonia was stored and protected within the mesopores. However, the ammonia synthesis rate for the mesoporous silica gyroid declined over time, which can be attributed to the incomplete desorption of ammonia during the “plasma-off” period. While the high surface area of the gyroid structure initially facilitates ammonia production, its tortuous morphology can slow the desorption kinetics. This slower desorption may result in ammonia being “trapped” within the pores, which could reduce ammonia synthesis rates over time, especially if the plasma-off period is not long enough to allow complete ammonia release. To verify this hypothesis, different “plasma-off” periods were explored. The results are presented in Figure S1. This figure shows that as the plasma-off time interval increases from 9 to 18 min, there is a more pronounced decrease in the ammonia detected during the plasma-off period. This suggests that with longer off times, the ammonia adsorbed on the material has more time to desorb before the plasma is turned on again. Further, longer plasma-off times (particularly in Figure S1d with an 18 min plasma-off period) appear to allow for conditions that lead to a higher ammonia synthesis rate during the subsequent plasma-on period when compared to shorter plasma-off times (as seen in Figure S1a with a 9 min plasma-off period). This is likely due to enhanced desorption, resulting in more available surface reaction sites when the plasma is turned back on.

Ammonia primarily undergoes physisorption on SiO_2_, allowing for desorption without the need for heating, thereby reducing the energy requirement and equipment cost (i.e., heating element). In previous work, we have studied this type of plasma adsorption–desorption phenomena over other chemistries.^46^ This reveals an important feature of customizing materials to enhance ammonia desorption without intensive energy requirements. Moreover, the sudden spike in the ammonia yield upon plasma shutdown may result from ammonia desorption from silica. However, further studies are essential to decoupling the effects of plasma-induced phenomena and thermal influences, enabling a deeper understanding of the experimentally underlying mechanisms.

### Optical Emission Spectroscopy Analysis

**Figure 4:**
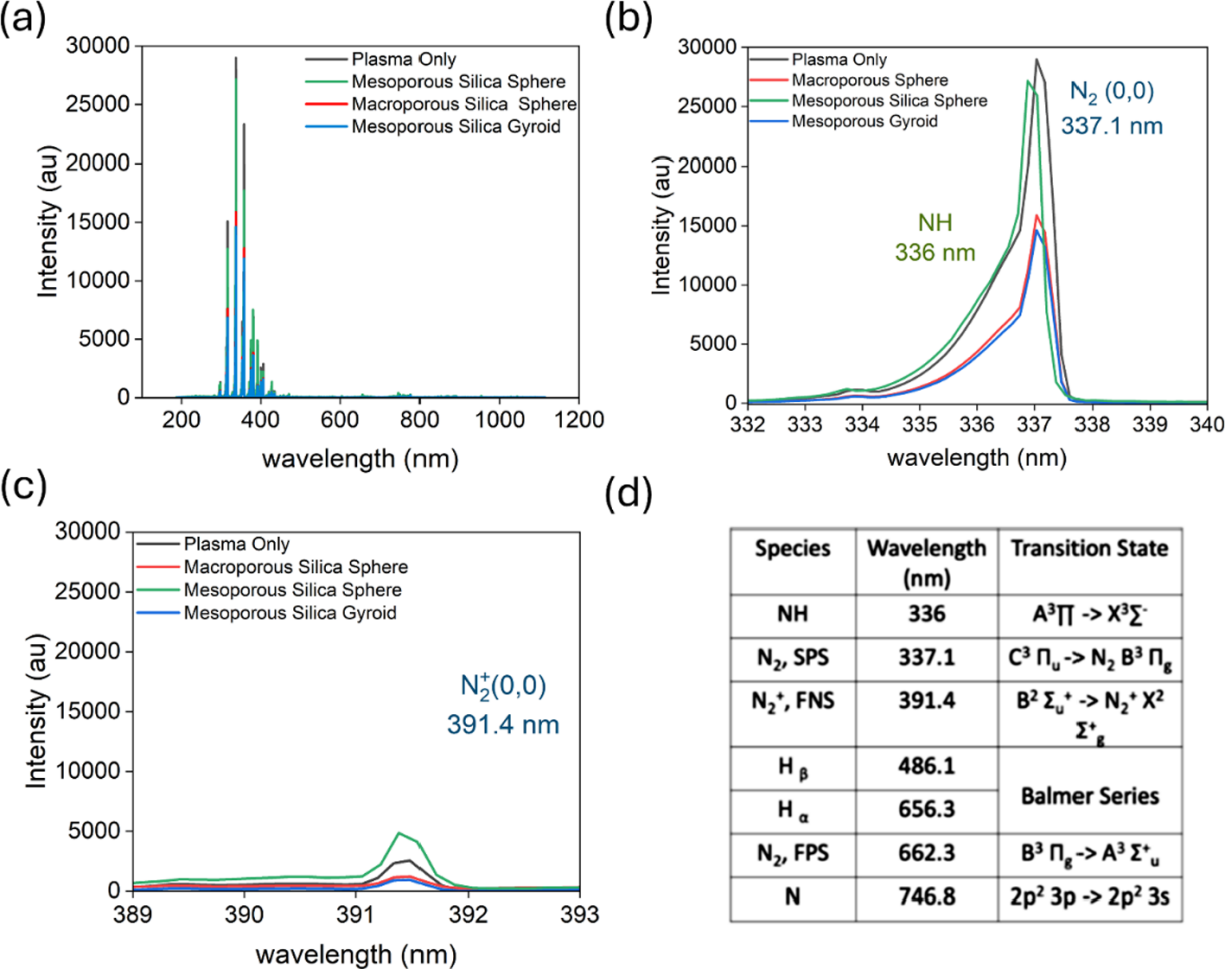
Emission spectra collected during plasma catalytic ammonia synthesis: (a) Comparative analysis for plasma only, mesoporous silica sphere, mesoporous silica gyroid, and macroporous silica sphere, 1:3 N_2_/H_2_ feed ratio at 15 W; (b) emission spectra of N_2_; (c) emission spectra of N_2_^+^; and (d) summary of important plasma species.

To gain a better understanding of the role of the plasma gas-phase species in the synthesis of ammonia, optical emission spectra were collected at a feed ratio of 1:3 (N_2_/H_2_) at 15 W. The OES spectra collected for plasma only, mesoporous silica sphere, mesoporous silica gyroid, and macroporous silica sphere during plasma reactions are shown in Figure 4. All species detected from the OES spectrum were consistent with our previous studies. The highest intensity peaks for N_2_ (337.1 nm) and N_2_^+^ (391.4 nm) were observed for the plasma-only test. However, Figure 4b–d shows the lowest intensity observed for the nitrogen and vibrational nitrogen species for the mesoporous silica gyroid. Optical emission spectroscopy reveals the gas-phase plasma species; however, in heterogeneous catalysis, reactions primarily occur on the material’s surface. Thus, the lower plasma intensity observed in mesoporous gyroids can be attributed to the increased interaction of plasma species with the gyroid surface. This serves as experimental evidence of the plasma–material synergism, demonstrating that the presence of the silica material plays a significant role in enhancing the synthesis of ammonia. Further, the hydrogen content of the macroporous silica sphere was the lowest when compared to the plasma only and other materials. The low intensity of Hα Balmer atomic species for the macroporous silica sphere suggests that hydrogen is recombining on the surface of the silica material and is evident of the silica’s effective ability to convert hydrogen to ammonia.

### Ammonia Synthesis Performance

**Figure 5:**
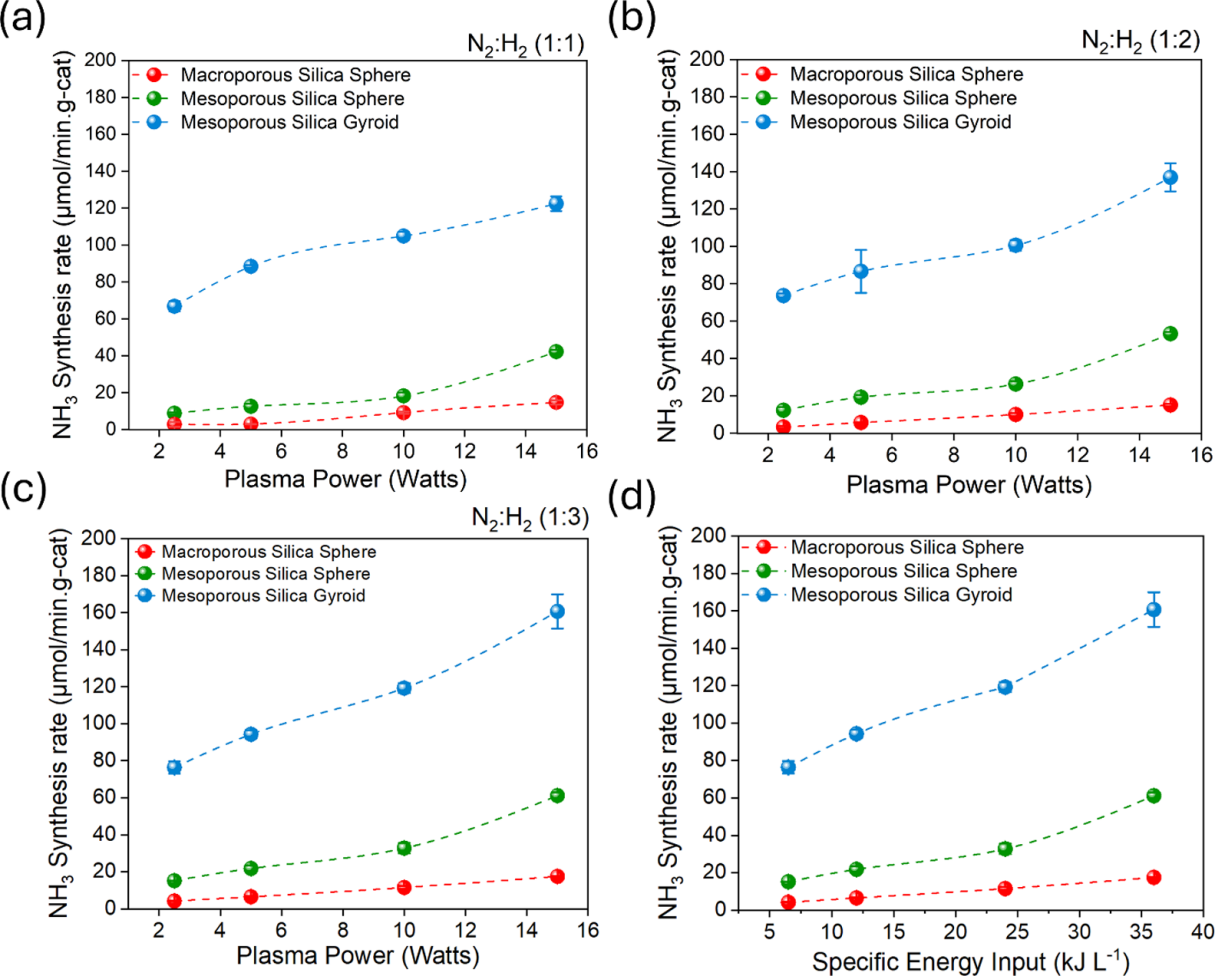
Ammonia synthesis rate (μmol/min·g-cat) produced from the (a) equimolar N_2_/H_2_ feed ratio of 1:1; (b) N_2_/H_2_ feed ratio of 1:2; (c) N_2_/H_2_ feed ratio of 1:3; and (d) specific energy input at 25 sccm of total flow rate.

In earlier research conducted by our group, we investigated three different pore regimes—nonporous, microporous, and mesoporous—and found that mesoporous materials were the most effective for ammonia synthesis.^31^ There was a trend in which the ammonia synthesis rate increased with the pore size regime, hence our motivation to investigate ammonia production over a macroporous silica sphere. The silica performance for plasma-driven ammonia production is shown in Figure 5a–c. The influence of the silica morphology and porous structure on the plasma-driven ammonia synthesis is thoughtfully addressed in this context. The mesoporous silica gyroid displays the highest ammonia production rate of 160.7 μmol/min·g of catalysis at a power of 15 W, followed by the mesoporous silica sphere with 61.2 μmol/min·g-cat. The reason for the better performance of the mesoporous silica gyroid is attributed to its unique morphology, which offers a higher surface area and facilitates better contact between the plasma discharge and the material surface compared to the sphere morphology. This enhanced interaction could lead to a higher concentration of reactive species generated within the pores, promoting ammonia synthesis. The H_2_-rich environment, for example, a nitrogen-to-hydrogen ratio of 1:3 (Figure 5c,d), slightly promotes ammonia production observed from all prepared materials shown in Figure 5. This aligns with prior research^29,31^ and is linked to a higher frequency of hydrogen radicals attaching to the material surface, which enhances ammonia production.

### Ammonia Energy Performance

**Figure 6:**
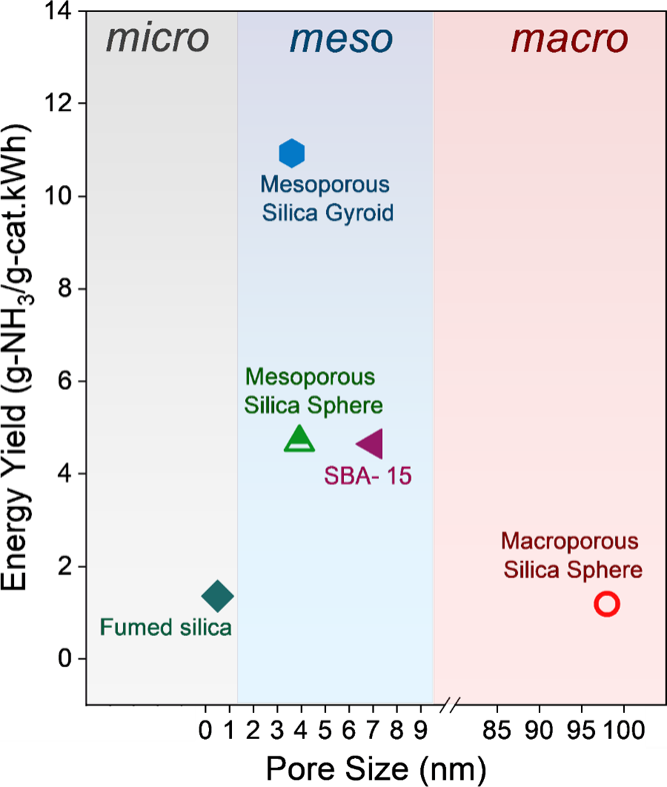
Comparison of ammonia energy yields obtained with various silica materials in this work with other materials: SBA-15^31^ and fumed silica.^31^

To deepen our understanding, we expanded our research to examine the ammonia energy yields associated with these materials. This study not only complements our findings on synthesis rates but also highlights the overall effectiveness of silicas in the synthesis of ammonia. Figure 6 compares the ammonia energy yield obtained with various silica materials in this work to other materials reported in the literature under similar reaction conditions. Each material contains 100% silica content. The highest ammonia energy yield of around 10.9 g-NH_3_/g-cat·kWh^,^ could be achieved when employing the mesoporous silica gyroids. As shown, the ammonia energy yield is the highest in the mesopore regime, confirming the influence of the porous structure and the morphology on ammonia energy yield, which can be related to the plasma–material synergism.

Mesoporous materials have a significantly higher surface area compared with their microporous and macroporous counterparts. This increased surface area can translate to enhanced diffusion of excited species into the pores and a higher area for surface reactions involved in ammonia synthesis. While macroporous materials can support a rapid transport of reactants within their pores, they lack the surface area needed for optimal ammonia synthesis. Further, the internal space of mesopores offers protection for the synthesized ammonia, preventing its decomposition by the plasma discharge and effectively shifting the equilibrium in the forward direction.^18^ These factors contribute to the enhanced reaction kinetics and higher overall ammonia energy yield of the mesoporous materials compared to those of the microporous and macroporous structures. Such findings demonstrate the potential of tailoring the morphology and porosity of a material to improve the ammonia energy yield.

To further illustrate these findings, we compared the energy yields at various plasma powers in Figure S2. Focusing on mesoporous morphologies allows for a deeper investigation into the optimization and feasibility of the best-performing material for NTP ammonia synthesis. It was observed that the mesoporous silica gyroid yielded 10.9 g-NH_3_/g-cat·kWh at 15 W compared to the mesoporous silica sphere (4.68 g-NH_3_/g-cat·kWh), an increase of 2.3 folds. The findings suggest that the mesoporous silica gyroid structure enhances interaction with the plasma field compared with the mesoporous silica sphere, leading to more effective utilization of energy input. The observed difference can be attributed to their distinct morphologies, despite both belonging to the same mesoporous regime. The gyroid structure offers a higher surface area and more entropic pore connectivity, which generates additional surface microdischarges, resulting in improved electrical field enhancement. Given this high energy yield, the mesoporous silica gyroid could be more readily integrated with renewable energy sources, such as solar or wind power, making it a promising candidate for decentralized ammonia synthesis applications.

### Cross-Pore Performance

#### Plasma-Induced Ammonia Desorption in Macroporous vs Mesoporous Silica

After investigating the pulsed on–off desorption method, we shifted our focus to the process of ammonia adsorption, followed by desorption facilitated by plasma treatment. Since mesoporous and macroporous silica represent two distinct pore regimes, the best-performing silica for NTP ammonia production from each type was selected to study the ammonia adsorption and desorption profiles.

For this analysis, ammonia was adsorbed on each silica for 30 min at a rate of 1 mL/min (1 sccm). The desorption was performed using helium (10 sccm) and a plasma power of 15 W for 5 min. By focusing solely on the adsorption characteristics, we can gain insights into how the structural differences of silicas affect their capacity to protect ammonia. This foundational understanding is crucial for optimizing materials for future applications in ammonia synthesis and intensified plasma-assisted processes. It should be mentioned that in this work, for the adsorption/desorption experiments, we used a 10% NH_3_/He gas mixture for 1 h of adsorption at standard temperature and pressure (STP). Desorption was performed using helium plasma at 15 W of power. Ammonia decomposition is possible due to dissociation energy thresholds: nitrogen (9.8 eV) > hydrogen (4.5 eV) > ammonia (4.3 eV). The binding energy between ammonia molecules and weakly acidic silanol groups (Si–OH–NH_3_) is 58 kJ/mol (0.5 eV).^51^ Based on these values, ammonia decomposition into NH_2_, NH, and related species is achievable to a certain degree under the conditions tested.

**Figure 7:**
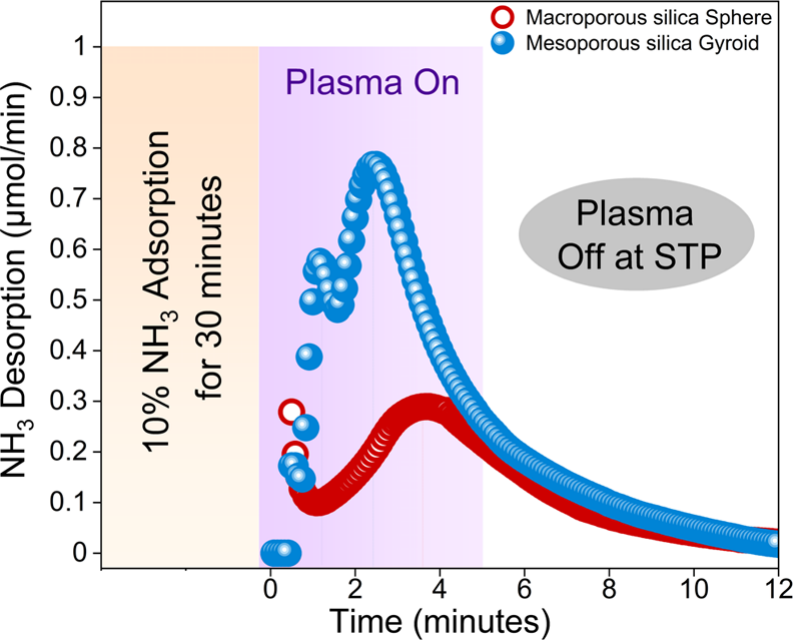
Comparison of adsorption–desorption profiles for the macroporous silica sphere and mesoporous silica gyroid.

As shown in Figure 7, the mesoporous silica gyroid has a significantly higher ammonia adsorption capacity compared with the macroporous silica sphere. The smaller pore size and complex, tortuous structure of the gyroid provide a greater surface area, resulting in a higher adsorption capacity. The desorption profile revealed two distinct peaks: an initial surface desorption at 1.1 min and a pore desorption at 2.5 min. These findings align with previous observations from our group on microporous molecular sieves,^36,46^ highlighting the impact of mesopore wall polarity on interactions with polar molecules. In contrast, the macroporous silica sphere shows a slower desorption rate, with a maximum of 3.6 min. This is likely due to its lower surface area and distinct isotherm behavior and pore size, both of which can impact desorption characteristics. Interestingly, the literature suggests that silica pore wall polarity influences significantly the adsorption and desorption characteristics, particularly through its interactions with polar molecules like water.^52^ Water interacts with both hydrophobic and hydrophilic sites within mesoporous walls, and its polarity, combined with its ability to form extensive hydrogen bonds,^52^ profoundly impacts these interactions. By comparison, ammonia, while also a polar molecule with a similar kinetic diameter (2.6 Å), exhibits weaker and less confined hydrogen bonding due to its single lone pair and three hydrogen atoms. In the context of plasma-induced ammonia desorption, surface polarity further affects the plasma interaction within the pore walls. Polar molecules, such as ammonia, experience field-induced distortions that influence desorption dynamics, with silanol (Si–OH) groups on hydrophilic pore walls, supporting a higher degree of hydrogen bonding, as evidenced by the ammonia desorption on silicas in this work at different pulsing intervals of time.

### Intrapore Performance

##### Effect of Different Morphologies on Discharge Properties for Mesoporous Silica

To further investigate the effect of morphology on electrical enhancement and, by extension, ammonia synthesis, we compare the electrical characterization of mesoporous gyroids and SBA-15. In previous studies, the unique doughnut structure of SBA-15 with hierarchically ordered mesopores made it an excellent candidate for NTP ammonia synthesis when compared to nonporous silica and fumed silica.^31^ SBA-15 has a BET surface area of 735.35 m^2^/g, making it comparable to the mesoporous silica gyroid with a surface area of 723.78 m^2^/g. The SBA-15’s doughnut-like morphology is shown in Figure S3. Notably, the mesoporous silica gyroid has more complex channels within its porous network, which is also evident in their respective isotherms (see Figure S4 for isotherm of SBA-15). Hence, it is a suitable candidate to investigate how variations in morphology influence electrical enhancement during plasma-assisted ammonia synthesis. This comparative analysis will provide insights into optimizing material design for enhanced performance in NTP ammonia synthesis.

**Figure 8:**
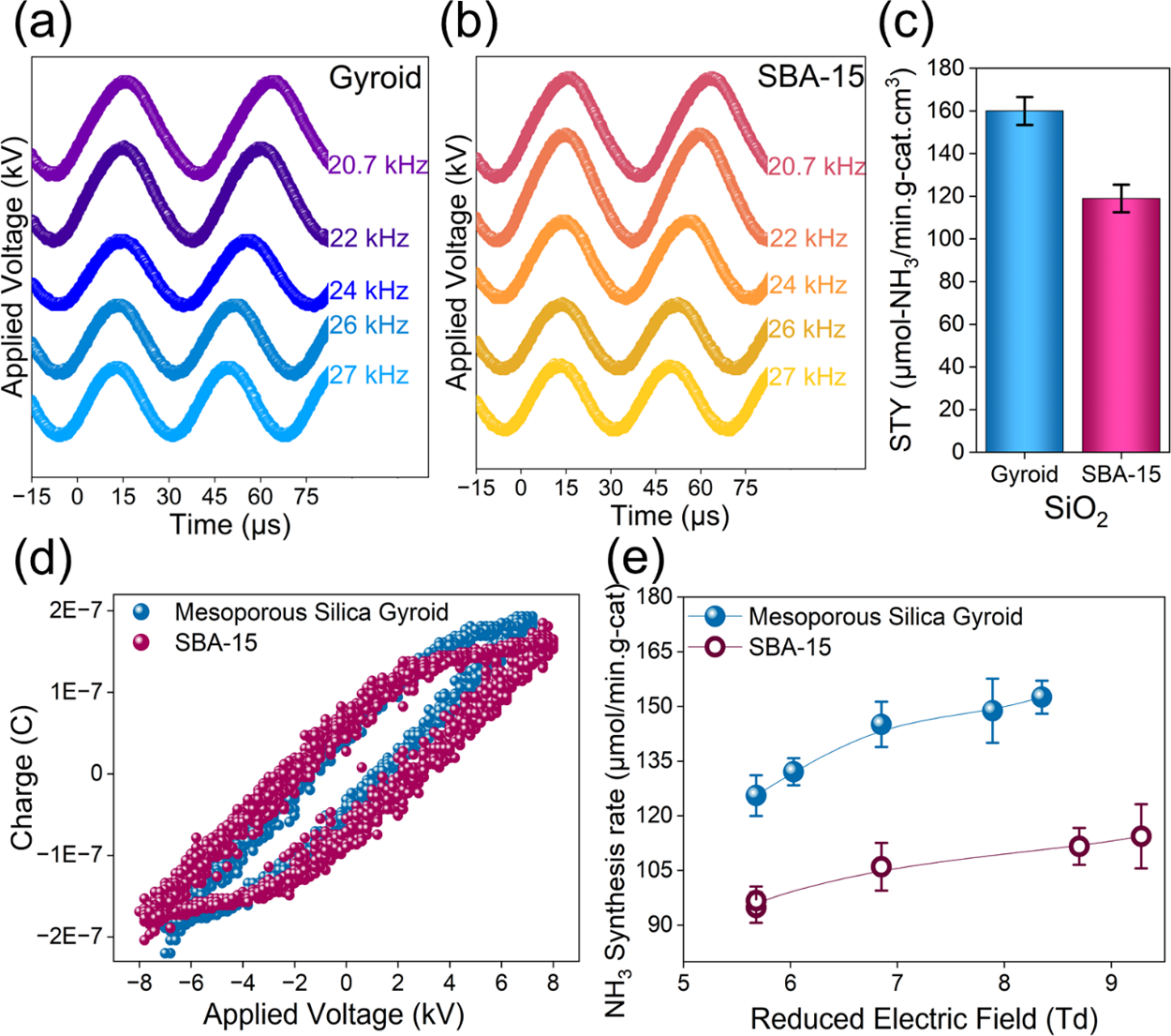
(a) Discharge profile of applied voltage vs. time (μs) of mesoporous silica gyroid, (b) discharge profile of applied voltage vs. time (μs) of SBA-15, (c) space–time yield (STY) of the ammonia synthesis rate (μmol/min·g-cat·cm^3^) for mesoporous gyroid vs SBA-15 at 22 kHz, (d) electrical characterization of the Lissajous curve (applied voltage vs. charge) at 22 kHz, and (e) performance range of the ammonia synthesis rate (μmol/min·g-cat) vs. E/N reduced electric field (Td).

We compared the performance of two different mesoporous morphologies: mesoporous silica gyroid and SBA-15 over a range of frequencies (20.7, 22, 24, 26, and 27 kHz). This range of frequencies tested was limited by the capabilities of our power supply. Figure 8a,b illustrates the effect of frequency on the ammonia synthesis rate. Based on the frequency experiments, we observed the highest performance at 22 kHz with a Vpk-pk of 15.2 ± 0.8 kV. At the same conditions, we compared the space–time yield (STY) for both materials, as demonstrated in Figure 8c, with ammonia generated per unit volume of reactor space and per unit time, based on the data the mesoporous silica gyroid outperformed SBA-15 by 1.34 fold, with a total of 160 μmol/min.g-cat.cm^3^ (see Figure 8c), indicating its superior catalytic performance in plasma-assisted ammonia synthesis.

Moreover, since both materials embed a similar composition (SiO_2_), Figure 8d shows the voltage–charge curve with a well-defined Lissajous curve for both materials under the same operating conditions. Based on the data, the voltage and frequency were kept constant for objective analysis, and the difference accounted for was minor; however, the mesoporous silica gyroid displayed a slightly higher charge than SBA-15, which is indicative of its Lissajous curve. Tailoring the morphology of materials can significantly enhance charge (Coulombs), directly correlating with improved ammonia synthesis rates. This variation is likely attributable to differences in the textural properties. To explore this further, we compared the E/N reduced electric field with ammonia synthesis rates and observed a clear connection, highlighting the critical role of material morphology in enhancing chemical reactivity. At 20.7 kHz and 14.70 W, the gyroid material shows an E/N of 7.5 Td, while SBA-15 at 13.65 W exhibits an E/N of 8.7 Td. This difference indicates that the gyroid material facilitates ammonia synthesis at a lower reduced electric field strength, likely due to its unique geometric structure, enabling localized electric field amplification. The observed geometric electric field amplification can be attributed to the gyroid’s interconnected pore structure, which enhances plasma–surface interactions and promotes efficient energy transfer. These effects reduce the required field strength while maintaining high ammonia synthesis rates. Detailed calculations on the reduced electric field (E/N) are provided in the Supporting Information. Interestingly, plasma exposure reduced the surface area of mesoporous silica gyroid and SBA-15 by 38.4% (723.78 to 445.69 m^2^/g) and 39.1% (735.34–447.47 m^2^/g), respectively, with a slight pore size increase from 3.26 to 3.4 nm for gyroid and 14.4–15.9 nm for SBA-15. These changes, consistent with previous findings on mesoporous silica,^25^ indicate slight mechanical wear after 5 plasma cycles at 15 W, without evidence of pore collapse (see Figure S5). Notably, across various ranges of the reduced electric field, mesoporous silica gyroid structures demonstrated superior ammonia synthesis rates (see Figure 8e). This can be attributed to their complex architecture, which may promote greater geometric field enhancement and higher relative permittivity over a wide frequency range (Figure S6), thereby facilitating more efficient nitrogen and hydrogen dissociation, as well as the generation of essential plasma species, ultimately driving higher ammonia production.

### Plausible Understanding toward Plasma Catalytic Ammonia Synthesis on Porous Materials

The experimental data facilitated an in-depth investigation into the effect of plasma catalysis on ammonia formation in porous materials, building on prior research that emphasized the significance of pore structure in catalytic performance.^15,25,30,31,42^ Previous studies by our group explored three distinct pore regimes—nonporous, microporous, and mesoporous—and identified mesoporous materials as the most effective for ammonia synthesis.^31^ These findings led to the exploration of a fourth regime: macroporous materials. The results revealed that macroporous silica (98 nm) exhibited lower ammonia synthesis compared to the mesoporous regimes. Furthermore, among the mesoporous structures, mesoporous silica gyroids (3.26 nm) demonstrated higher ammonia synthesis and energy efficiency than SBA-15 (7 nm) and mesoporous silica spheres (3.92 nm), indicating that morphology plays a critical role. To understand the underlying mechanisms, the investigation was approached from two perspectives: (1) plasma propagation and electrical enhancement and (2) in situ polarizability and surface interactions between ammonia and silanol (Si–O–H) groups. These perspectives collectively provided a comprehensive understanding of the observed catalytic behavior.

First, the mesoporous silica sphere, characterized by its smooth and uniformly curved surface, possesses a consistent radius of curvature. When subjected to an external electric field, the electric field distribution remains relatively uniform across the sphere’s surface due to the absence of sharp edges or points, thereby minimizing localized electric field concentration. On the other hand, SBA-15 is a torus, and the curvatures are generally not the same in all directions, leading to major and minor radii. In contrast, a mesoporous silica gyroid structure exhibits an entropic, triply periodic geometry with regions of varying radii of curvature. The gyroid’s surface alternates between sharp curves with small radii of curvature and gentler curves with larger radii of curvature. When an electric field is applied to a gyroid, the field intensity significantly increases in areas with smaller radii of curvature (sharp curves), leading to a localized concentration of the electric field.^40,53^ Thus, the gyroid structure was more effective in promoting plasma-enhanced processes due to the enhanced electric fields and improved plasma distribution within its network. In cases where the pore opening was significantly smaller than the Debye length, plasma propagation into the pore was highly restricted as pores ranging from 3 to 98 nm—much smaller than the typical Debye length of 2.5 μm in DBD plasma; the electron density inside the pore remained extremely low, leading to limited photoionization but still generating a flux of photons, which propagate inside the pores to seed plasma-induced species, as described by Mark Kushner’s group^53−55^ and observed experimentally in this work with macroporous silica with a 98 nm larger pore opening compared to mesoporous silica.

**Figure 9:**
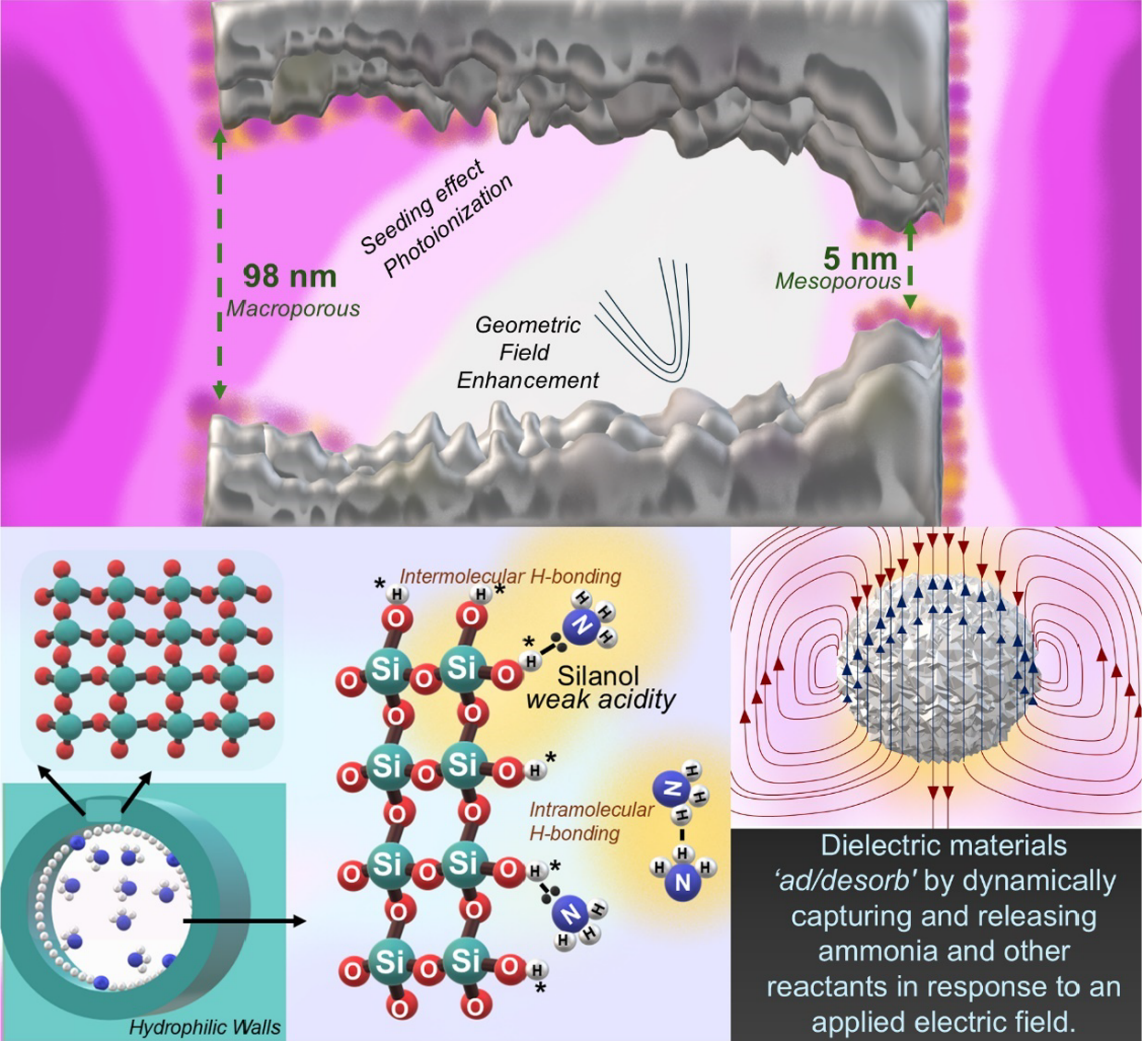
Plausible ammonia synthesis pathway in porous regimes.

Moreover, ammonia has higher polarizability (2.8 Å^3^) compared to nitrogen (1.7 Å^3^) and hydrogen (0.8 Å^3^), making it prone to significant distortion of its electron cloud in the presence of an electric field and leading to an induced dipole moment. When the reactor chamber is packed, this could lead to improved ammonia adsorption on silica.^31,56^ In plasma catalysis, nitrogen (N_2_) and hydrogen (H_2_) decompose into radicals, specifically atomic nitrogen (N) and atomic hydrogen (H).^1,57^ In the presence of silica with a surface-containing electronegative oxygen species, the high concentration of adsorbed hydrogen atoms could lead to the formation of silanol groups (SiOH). These silanol groups are weakly acidic in nature, which could further enhance the adsorption of basic molecules like ammonia.^51,58^ The polarizability of ammonia allows the distribution of its charge, specifically on a lone pair, facilitating the formation of an intramolecular bond between ammonia and the silica surface. During continuous plasma operation, catalytic reactions eventually reach steady-state equilibrium. However, by using plasma pulsing (on/off cycles), this equilibrium can be disrupted, allowing for controlled energy input that overcomes typical reaction limits. Plasma pulsing facilitates ammonia formation and desorption at atmospheric pressure and room temperature, all without significant bulk heating. By switching the plasma on and off, the system minimizes excess energy input, which favors ammonia formation while reducing its decomposition. In this process, the interaction of electric fields with rigid dielectric materials, such as silica, can be visualized as being dielectric-controlled. These series of events allow the dielectric materials to “adsorb and desorb,” dynamically capturing and releasing ammonia and reactants in response to the applied electric field (see Figure 9). Based on the literature, the silicates exhibit unique dielectric responses with distinct peaks (boson peak at 2 THz and Si–O–Si ionic resonance at 13 THz), related to structural features like curvature and bond angles.^59^ These properties contribute to geometric field enhancement and intensify field distortion, thereby boosting polarization effects. The Si–O–Si bond’s ionic resonance also adds flexibility, as the strength of the electric field influences silica’s dielectric properties, enhancing its capacity to adsorb ammonia and enabling controlled desorption within the plasma environment.

## Conclusions

In this work, plasma-driven ammonia production over silicas with various morphologies, including spherical and gyroid, and different porous regimes, such as mesoporous and macroporous, was investigated. The influence of silica morphology and porosity on plasma-powered ammonia production was investigated thoughtfully. The ammonia production rate was favored in the following order: mesoporous silica gyroid > mesoporous silica sphere > macroporous silica sphere. The mesoporous silicas offer better plasma-active species diffusion to boost the formation of ammonia, attributed to their morphology. Mesoporous silica gyroid was favorable for obtaining a higher ammonia production rate given their better plasma–material synergism due to geometric field enhancements and limited in situ ammonia decomposition. The findings from this work represent important steps toward developing earth-abundant materials by tailoring their morphology and porous structure to enhance the plasma ammonia production yield.

## Data Availability

A reasonable request to the corresponding authors may permit the data used to support the results of this study.

## Supporting Information Available

Plasma catalytic pulsing (plasma on/off) for mesoporous silica gyroids; ammonia energy performance for all employed materials; pore structure of SBA-15 assessed by scanning electron microscopy (SEM); textural properties of SBA-15; methods and equations employed in this work; isotherm comparison after plasma exposure; and relative permittivity (an approximation) (PDF)

## Notes

All experiments in this study were carried out in adherence to ethical standards, and no ethical concerns were encountered during the experiments or preparation of this manuscript.
