# Supplementary material for: Cold-Plasma-Driven Ammonia Synthesis over Porous Silica: The Role of the Morphology
Source: ACS Mater Au. 2025 Jan 14;5(2):385–96. doi: 10.1021/acsmaterialsau.4c00159 (PMC11907291; doi:10.1021/acsmaterialsau.4c00159)
Supplement: Supplementary file 1 — mg4c00159_si_001.pdf [file mg4c00159_si_001.pdf]

Supporting Information

Cold plasma driven ammonia synthesis over porous silica: The role of the morphology

Fnu Gorky[a], Vashanti Storr[a], Jacek B. Jasinski[b], Maria L. Carreon[a,]*

[a]Ralph E. Martin Department of Chemical Engineering, University of Arkansas, 3202 Bell Engineering Center Fayetteville, AR 72701-1201, USA.

[b]Conn Center for Renewable Energy Research, JB Speed School of Engineering, University of Louisville, Louisville, KY 40292, USA.

Corresponding authors

*mc138@uark.edu

Table of Contents

- Plasma catalytic pulsing (Plasma On/Off) for mesoporous silica gyroid……..**(Page S2)**
- Ammonia Energy Performance for all employed materials…………………...**(Page S3)**
- Pore structure of SBA-15 assessed by Scanning Electron Microscopy (SEM).**(Page S4)**
- Textural properties of SBA-15………………………………………………...**(Page S5)**
- Methods and Equations employed in this work………………………………..**(Page S6)**
- Isotherm comparison after plasma exposure……………………………………..(**Page S9)**
- Relative Permittivity (An approximation)...……………………………………..**(Page S10)**
- References……………………………………………………………………**(Page S13)**

Plasma catalytic pulsing (Plasma On/Off) for mesoporous silica gyroid

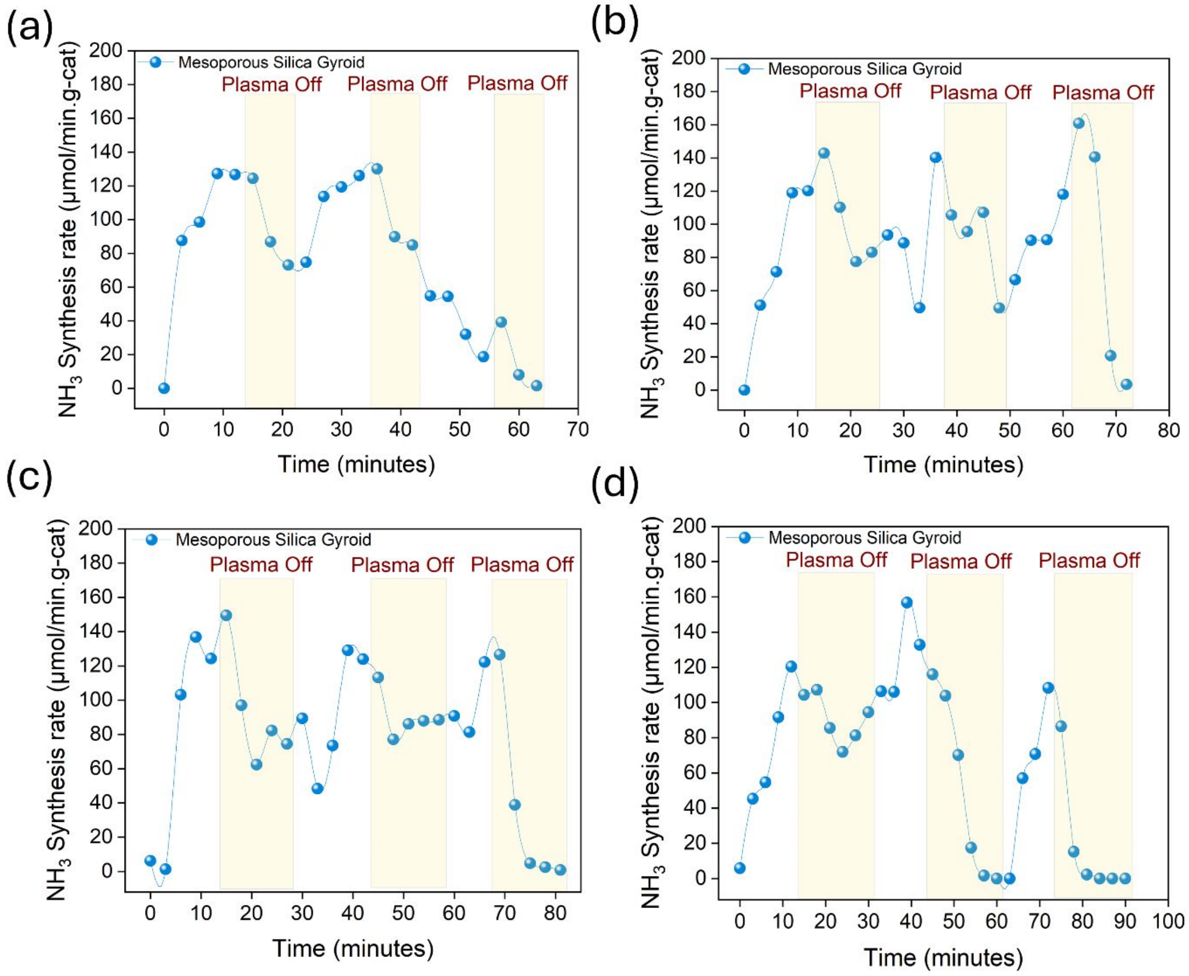

Figure S1. Plasma catalytic pulsing (Plasma On/Off) for fresh mesoporous silica gyroid, at hydrogen rich feed (1:3) N_2:H_2, 25 sccm total flow rate, and 15 ± 0.2W plasma power, **(a)** 9 min plasma off period, **(b)** 12 mins plasma off period; **(c)** 15 mins plasma off period; **(d)** 18 mins plasma off period.

Ammonia Energy Performance for all employed materials

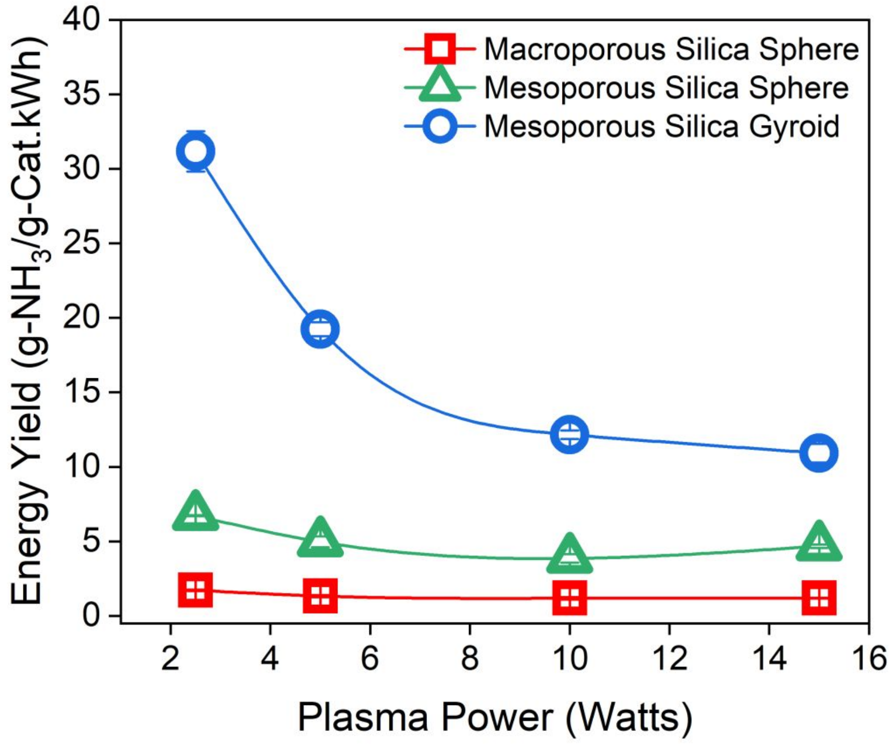

Figure S2. A comparison ammonia energy yield obtained with mesoporous silica sphere, mesoporous silica gyroid and macroporous silica sphere.

Pore structure of SBA-15 assessed by Scanning Electron Microscopy (SEM)

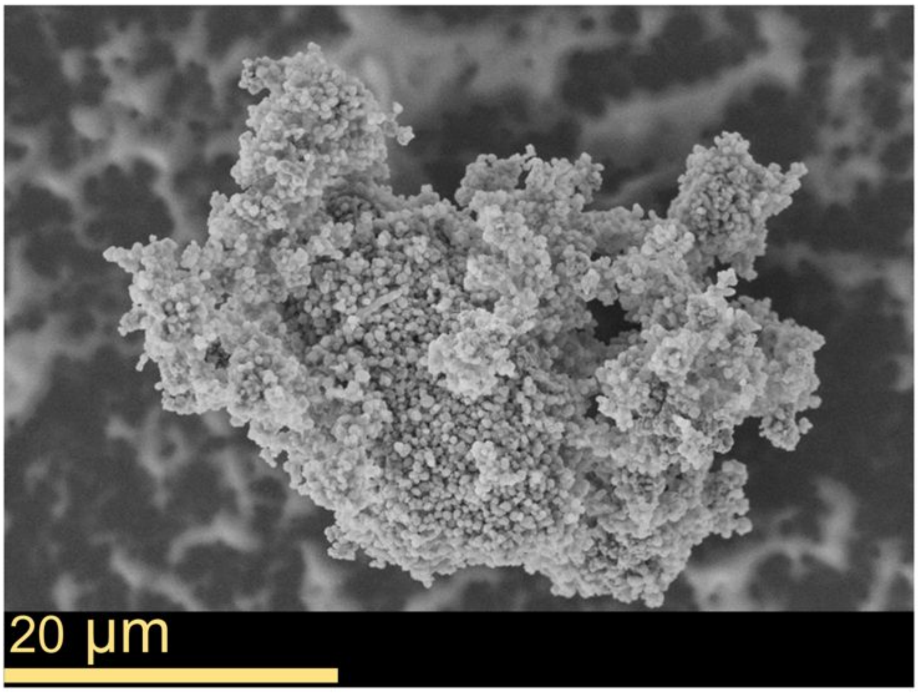

Figure. S3 SEM images for the fresh SBA-15.

Textural properties of SBA-15

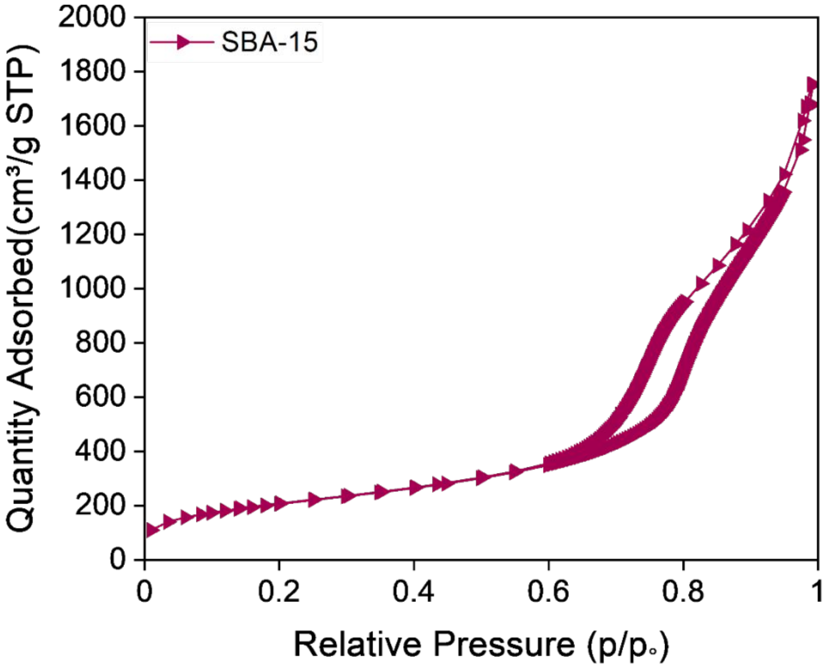

Figure S4. N_2 adsorption-desorption isotherm curves of fresh SBA-15.

Methods and Equations employed in this work

1. Plasma Power (Watts)

$$\textbf{Power}_{\textbf{avg}} = \oint v(t) * q(t) * d(t) = \frac{f}{2\pi} * S$$

- $Power_{avg}$ is the average power in W,

- $v(t)$ is the voltage measured by the oscilloscope,

- $q(t)$ is the charge measured by the oscilloscope in C,

- f is the frequency in Hz,

- S is the area under the curve for one cycle.

Units: Watts

2. Specific Energy Input (kJ/L)

$$\frac{Plasma\ Power\ (kW)}{Q\ (Flow\ rate)\ (sccm\ or\ ml/minute)} * 60(second/minute) \quad \text{Units: } \frac{kJ}{Litre}$$

3. NH_3 Synthesis rate

$$\frac{GC\ area\ * 0.745\ (micromoles/sccm * s)}{Calibration\ factor}$$

From Equation:

- NIST Conversion factor (sccm to mol/s)

Gas Flow: 1 sccm = 7.45E-7 mol/s

- 1 mol= 1000000 micromoles

- (7.45E-7 mol/s) (1000000micromoles/1mol) = 0.745 micromoles/s

Units: $\frac{micromoles}{seconds}$

4. **Discharge Gap:**

Quartz Tube Outer Diameter (O.D.) = 6.35 mm

Quartz Tube Inner Diameter (I.D.) = 4 mm *(radius = 2 mm)*

Inner Electrode (Tungsten) (O.D.) = 2.4 mm *(radius = 2.2 mm)*

Discharge gap = Radius of Inner Quartz tube – Radius of Inner Electrode (2.2 mm - 2mm)

Discharge gap = 0.8 mm

5. **Residence Time:**

Details for residence were also previously reported from our group[1].

Volume of the reactor = 1.29 ml

Volumetric Flow rate (sccm or ml/min) = 25 ml/min

Void Volume of the reactor = 1.159 ml

Residence time = $\frac{Void\ volume\ of\ the\ reactor}{Q\ (volumetric\ flow\ rate)} = \frac{1.159\ ml}{25\ ml/min} = 0.04636\ min * 60\ \left(\frac{s}{min}\right) = \mathbf{2.78}\ s$

Residence time = $2.78\ s$

Reduced Electric Field (E/N) in Townsend

To calculate the electric field during plasma catalytic operations, we analyzed experimental data collected from the oscilloscope. The maxima and minima of the applied voltage within the observed time frame were identified and averaged.

For instance, the maximum voltage recorded was +7.6 kV, and the minimum was −7.4 kV, giving a **peak-to-peak voltage (Vpk-pk)** of 15 kV.

Using this value, the electric field (E) can be calculated as follows:

E (V/m) = $\frac{\mathrm{Voltage_{pk-pk}}}{\mathrm{reactor\ length\ (d)}}$

where d is the reactor length where the discharge occurs. For a reactor length of 70mm (outer mesh length of the ground electrode), the electric field is:

Electric Field = (15000 Volts)/ (0.07 m) = 214285.7 V/m

Number Density of Gas (N):

Number density of gas is calculated using the ideal gas law:

Number Density of Gas (N) = $\frac{\text{Pressure (P)}}{\text{Boltzmann constant } (k_B) * \text{Temperature(K)}}$

Where:

P is the pressure (101325 Pa), since the plasma catalytic operation was conducted at atmospheric pressure.

$\mathbf{k_B}$ is the Boltzmann constant (1.38×10^{-23} J/K)

*Note: 1 Joule = 1 Pa*m*^{3}

T is the temperature, 298.15 K, since experiments were carried out at room temperature without any external heating.

Number Density of Gas (N) = $\frac{101325 \text{ Pa}}{1.38\times10^{-23} \text{ Pa.m}^3/\text{K} * 298.15 \text{ K}}$ = 2.46×10^{25}m−3

Calculating Reduced Electric Field (E/N) = $\frac{214285.7 \text{ V/m}}{2.46\times10^{25}\text{m}^{-3}}$ = 8.70×10^{21} V.m^2

Since E/N is expressed in Townsend (Td)

Note: 1 Td = 10^{-21} *V.m*^{2}

E/N = (8.70×10^{21} V.m^2) ***** (10^{-21} V.m^2) **= 8.70 Td**

Isotherm comparison after plasma exposure

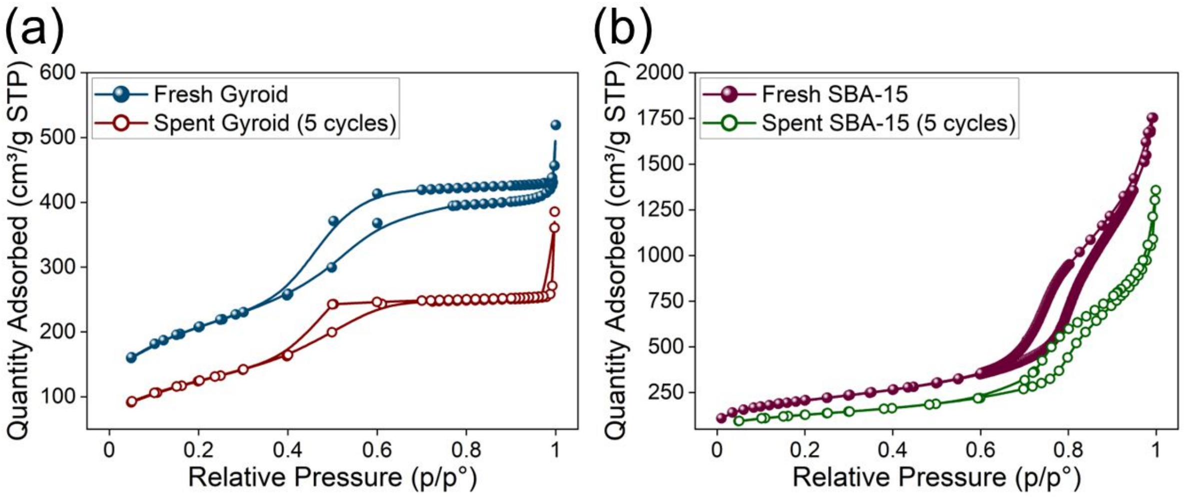

Figure S5. N_2 adsorption-desorption (a) mesoporous gyroid and (b) SBA-15 isotherms of fresh and spent after 5 cycles of DBD plasma exposure at 15 W plasma power. *Note: The Y-axes differ to account for variations in the quantity adsorbed, ensuring optimal visual comparison between the two plots.*

Relative Permittivity: An approximation

The relative permittivity data for mesoporous silica significantly deviates from the dielectric constant of pure silica (ϵ_r=3.9)[2, 3], particularly at lower frequencies. At 20.7 kHz, the relative permittivity of mesoporous gyroid is 10.70, while that of SBA-15 is 8.16. Although both materials share the same chemical composition, the geometrical structure plays a critical role, with the gyroid exhibiting higher relative permittivity due to its triply periodic geometry. At 22 kHz, the relative permittivity further increases to 12.48 for the gyroid and 8.79 for SBA-15. Beyond 24 kHz, the relative permittivity decreases rapidly, approaching values closer to pure silica at 27 kHz (ϵ_r = 4.39 for the gyroid and ϵ_r = 4.11 for SBA-15). This trend can be attributed to Maxwell-Wagner-Sillars (MWS) polarization[4], a phenomenon common in heterogeneous materials like porous silica. At lower frequencies, interfacial polarization occurs due to the accumulation of charges at the interface between the silica matrix and the pores[4]. The lower frequencies could allow necessary time for charge migration and accumulation, leading to a pronounced increase in relative permittivity. The higher relative permittivity of the mesoporous gyroid compared to SBA-15 is attributed to its triply periodic structure, which improves the geometric field enhancement.

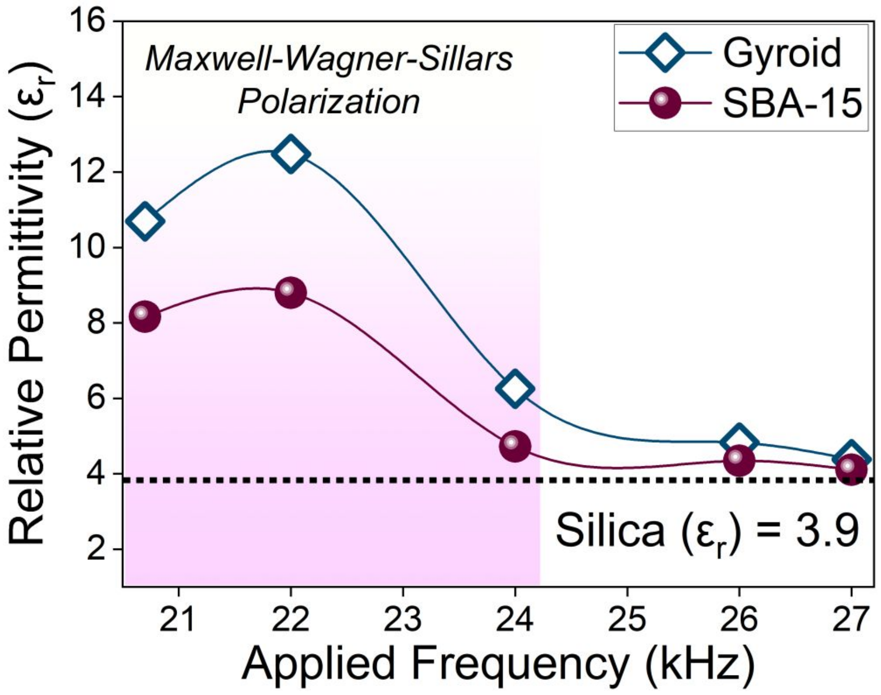

Figure S6. Comparison on Applied frequency Vs. Relative permittivity (ϵ_r) for mesoporous gyroid and SBA-15 during plasma catalytic operation.

Calculating relative permittivity (ε_r) : An Approximation

The detailed equation for estimating the relative permittivity is widely reported in literature for porous solids[5, 6]. In this study we aimed to approximate the relative permittivity (ε_r) during plasma catalytic operations by analyzing experimental data collected from an oscilloscope. In the literature the relative permittivity silica has been widely studied[7]. Plasma power (Watts) was estimated experimentally, as detailed in the previous section. The **electrode gap (d)**, in meters, was determined experimentally based on the packed area between the live electrode and the quartz tube. As displayed in the equation below, the numerator indicates the energy input (plasma power) and effective path length for the electric field (electrode gap). The **angular frequency (ω)**, expressed in radians per second, was calculated using the operating frequency of the plasma power, which for this study ranged between 20 and 27 kHz. The **permittivity of free space (ε_0)**, a fundamental constant in vacuum, was taken as 8.854×10^{-12} F/m. The **cross-sectional area (A)**, measured in square meters, was derived from the volume of the packed bed divided by the length of the packed area. The **peak-to-peak voltage** was determined experimentally. The denominator describes the electric field strength (voltage), energy dissipation (angular frequency), and spatial distribution of the polarization (across the cross-sectional area). For this study, the value of the **loss tangent (tanδ)** was adopted from the literature[4]. Loss Tan represents the ratio of imaginary and real parts of materials relative permittivity. Since the range dielectric barrier discharge (DBD) operated in kHz frequency range (*power supply limitations*), the reported value for pure silica was tanδ = 0.12. The calculation is based on the following formula

$$\textbf{Relative permittivity } (\varepsilon_r) = \frac{2.(P).(d)}{(\omega).(\varepsilon_0).(A).(V_{pk-pk})^2.\,(tan\delta)}$$

Note, since relative permittivity is dimensionless, we have provided unit conversions for each parameter below:

P: Plasma Power (Watts), Watts = Joules/sec = $(Kg.m^2)/(s^3)$

d: Electrode gap (meters), m

ω: Angular frequency (radians/sec) = (1/s)

ε_0: Permittivity of free space (F/m), (1F = 1C/1V) = $(s^4.A^2)/(m^3.Kg)$

A: Cross-sectional area (m^2)

V_{pk-pk}: Peak-to-peak voltage, V = $(Kg.m^2)/(s^3.A)$

tanδ: Loss tangent (dimensionless)

Note: For this work, the value of tanδ was taken from the literature[4]. Since the dielectric barrier discharge (DBD) operations in this study were in the kHz frequency range, the reported value for pure silica is tanδ=0.12

Unit Conversion: **Relative permittivity (ε_r) =** $\frac{\left(\frac{\mathrm{Kg.m^2}}{\mathrm{s^3}}\right).(m)}{(\frac{1}{s}).(\frac{\mathrm{s^4.A^2}}{\mathrm{m^3.Kg}}).(\mathrm{m^2}).\left(\frac{\mathrm{Kg.m^2}}{\mathrm{s^3.A}}\right)^2} = \frac{(\frac{\mathrm{Kg.m^3}}{\mathrm{s^3}})}{(\frac{1}{s}).(\frac{\mathrm{s^4.A^2}}{\mathrm{m^3.Kg}}).(\mathrm{m^2}).(\frac{\mathrm{Kg^2.m^4}}{\mathrm{s^6.A^2}})}$

$= \frac{(\frac{\mathrm{Kg.m^3}}{\mathrm{s^3}})}{(\frac{\mathrm{Kg.m^3}}{\mathrm{s^3}})} = 1$ ***(dimensionless)***

References

(1) Shah, J. R.; Gorky, F.; Lucero, J.; Carreon, M. A.; Carreon, M. L. Ammonia Synthesis via Atmospheric Plasma Catalysis: Zeolite 5A, a Case of Study. *Industrial & Engineering Chemistry Research* **2020**, *59* (11), 5167-5176. DOI: 10.1021/acs.iecr.9b05220.
(2) Volksen, W.; Miller, R. D.; Dubois, G. Low dielectric constant materials. *Chemical reviews* **2010**, *110* (1), 56-110.
(3) Cardona, M.; Paul, W.; Brooks, H. Dielectric constant of germanium and silicon as a function of volume. *Journal of Physics and Chemistry of Solids* **1959**, *8*, 204-206.
(4) Hussain, W. A.; Hussein, A. A.; Khalaf, J. M.; Al-Mowali, A. H.; Sultan, A. A. Dielectric properties and AC conductivity of epoxy/alumina silicate NGK composites. *Advances in chemical engineering and science* **2015**, *5* (3), 282-289.
(5) Revil, A. Effective conductivity and permittivity of unsaturated porous materials in the frequency range 1 mHz–1GHz. *Water resources research* **2013**, *49* (1), 306-327.
(6) Robinson, D. A.; Friedman, S. P. A method for measuring the solid particle permittivity or electrical conductivity of rocks, sediments, and granular materials. *Journal of Geophysical Research: Solid Earth* **2003**, *108* (B2).
(7) Jain, A.; Rogojevic, S.; Ponoth, S.; Agarwal, N.; Matthew, I.; Gill, W. N.; Persans, P.; Tomozawa, M.; Plawsky, J. L.; Simonyi, E. Porous silica materials as low-k dielectrics for electronic and optical interconnects. *Thin Solid Films* **2001**, *398*, 513-522.
